# Genetic Variability for Micronutrient Content and Tuber Yield Traits among Biofortified Potato (*Solanum tuberosum* L.) Clones in Ethiopia

**DOI:** 10.3390/plants12142625

**Published:** 2023-07-12

**Authors:** Ebrahim Seid, Lemma Tessema, Tesfaye Abebe, Atsede Solomon, Abebe Chindi, Betaw Hirut, Kasaye Negash, Egata Shunka, Zewditu Mogse, Gabriela Burgos, Thiago Mendes

**Affiliations:** 1 Ethiopia Institute of Agricultural Research, Holetta Agricultural Research Centre, Addis Ababa P.O. Box 2003, Ethiopia; lematessema@gmail.com (L.T.); destaadera@gmail.com (T.A.); abechindi@gmail.com (A.C.);; 2 School of Integrative Plant Science, Plant Pathology and Plant-Microbe Biology (PPPMB) Section, Cornell University, Geneva, NY 14456, USA; 3 International Potato Centre (CIP), Lima 00051, Peru; g.burgos@cgiar.org; 4 International Potato Centre (CIP), Nairobi 00100, Kenya

**Keywords:** correlation, heritability, genetic variation, breeding and cluster analysis

## Abstract

**Simple Summary:**

Almost 2 billion people worldwide, especially in developing countries, are suffer due to micronutrient deficiencies, sometimes known as “hidden hunger”. In Ethiopia, diet-related iron and zinc deficiency is a significant public health issue. The potato has the potential to be a significant iron and zinc source. A total of 45 potato genotypes, including the variety Gudanie, were grown in field trials in a 9 × 5 alpha lattice design with three replications. In the present study, high heritability estimates along with high genetic advance as percent of mean were obtained for average tuber number, tuber yield, and Zn concentration. The broad-sense heritability for Fe and Zn concentrations found in the tetraploid population is lower than in diploid potato clones. Negative correlations and direct effects on most of the traits with Fe and Zn contents. Therefore, attaining simultaneous genetic gain for yield and enhanced Fe and Zn concentrations will be challenging. Cluster II contained the most prominent genotypes, having better mean values compared to all other genotypes for Fe and Zn. In conclusion, more sites, including more genotypes, are needed to find a variety with high Fe and Zn contents and while taking into good tuber yield.

**Abstract:**

Malnutrition is one of the global issues of public health concern, and iron and zinc deficiencies are at the top of the list. Iron deficiency affects more than 2 billion people in the world and is a major cause of anemia. Potato has the potential to be an important source of iron and zinc. This study assessed the nature and magnitude of genetic variability in Fe and Zn concentrations, tuber yield, and quality traits among biofortified tetraploid potato clones and their relationships through correlation and path analysis. A total of 45 potato genotypes, including the variety Gudanie, were grown in field trials in a 9 × 5 alpha lattice design with three replications. Significant differences in mineral, tuber quality, and yield traits were observed among the genotypes, and high broad-sense heritability was obtained for most traits, suggesting that progress through breeding can be achieved. However, negative correlations and direct effects on most of the traits with Fe and Zn contents are found both at genotypic and phenotypic levels. Therefore, attaining simultaneous genetic gain for yield and enhanced Fe and Zn concentrations will be challenging. Cluster analysis assembled them into five groups. Cluster II contained the most prominent genotypes, having better mean values compared to all other genotypes for micronutrient traits, viz., Fe (23.80 mg kg^−1^) and Zn (17.07 mg kg^−1^). The results of this study confirm the presence of sufficient genetic variation for iron and zinc mineral concentration and the possibility to make significant progress through breeding.

## 1. Introduction

Potato (*Solanum tuberosum* L.) is the most important crop, versatile worldwide, and central to global food security and nutrition [1,2]. Around the world, nearly 2 billion people, mostly in developing countries, suffer from micronutrient malnutrition, often referred to as ‘hidden hunger’ [3,4]. The most common micronutrient deficiencies in women are iron, vitamin A, iodine, and zinc [5]. Iron (Fe) and zinc (Zn) deficiencies in diets are a major public health problem in Ethiopia [6,7,8]. Fe deficiency in Ethiopia is reported among preschool-age children (30%), school-age children (20%), and women of reproductive age (16%), whereas the prevalence of Zn deficiency is about 35% in preschool-age children, 36% in school-age children, and 34% in non-pregnant women of age 15–49 [6]. Potato’s importance as a food crop has been increasingly expanding over the years in Ethiopia [9]. Fe found in potato is very well absorbed by the human body. According to recent Fe absorption studies in women using stable isotopes, the Fe from yellow-fleshed biofortified potatoes has a remarkably high absorption (14–29%); this is greater than that reported for other biofortified crops such as pearl millet and beans (4 to 8%, respectively) [10,11]. In another related research, the higher bioavailability of minerals in potato tuber and other major food crops is reported owing to the high concentrations of promoter chemicals such as ascorbic acid that promote micronutrient absorption by the body [12] and low level of inhibitor chemicals [13].

Various research studies have been conducted around the world, including in Ethiopia, on assessing the concentrations of Fe and Zn in potato. There are significant differences in agronomic, Fe, and Zn micronutrient traits among widely grown farmer varieties in Ethiopia and improved tetraploid clones introduced by the International Potato Center (CIP) Lima, Peru, to Ethiopia [8]. More importantly, one variety named ‘Feyisa’ with better Fe and moderate levels of Zn was released for use by growers and consumers in 2020 from the tetraploid clones introduced by the International Potato Center (CIP) through the HarvestPlus project. Genetic variability in the Fe concentration of potato tubers ranges from 30 to 185, 11.71 to 131.05, 29.87 to 157.96, and 48.85 to 122.69 mg kg^−1^, respectively, on a dry-weight basis [14,15,16,17]. In a previous study, a much wider range of concentrations for Fe and Zn was reported in Ethiopia on a dry-weight basis [18]. As pointed out, the observed result is a promising opportunity for the breeding program in Ethiopia to increase the bioavailable Fe and Zn concentrations of potato tubers as the heritability of Fe concentration in potato is moderately high [19].

Studying the genetic variability present among different potato genotypes for a given character is a basic precondition to designing systematic breeding methods [20]. The genetic variability and breeding strategies in potato for essential micronutrients, such as Fe and Zn, have been reported by different authors [16,21,22,23]. Investigating the correlation among characters is vital for any improvement program. The correlation coefficient measures the relationship between two variables and measures the rate of change in dependent variable per unit rate of change in independent variable and varies between +1 and −1. However, these figures alone tell us nothing about the causal relation of variables [24]. Hence, path coefficient analysis is relevant to identify the direct and indirect causes of the associations of characters with each other and to measure the relative importance of each [25]. Path analysis is the portioning of the total correlation into direct and indirect effects of the independent variable(s) on the dependent variable [26].

Diversity in plant genetic resources provides an opportunity for plant breeders to develop new and improved cultivars with desirable characteristics, which include both farmer- and breeder-preferred traits. Genetic diversity facilitates breeders to develop varieties for specific traits such as quality improvement and tolerance to biotic and abiotic stresses [27]. Cluster analysis and principal component analysis (PCA) are also frequently used statistical tools for exploring genetic diversity while securing relative basic differences among study samples. Cluster analysis is a classification method, which is used to explore similarities and diversity in a collection of study subjects [28].

In Ethiopia, more than 35 improved potato varieties have been released by different research centers and institutions since the inception of potato research and development programs with no emphasis on nutrition. Hence, information on the Fe and Zn contents of such released varieties is scarce, and only a few studies have been conducted on the micronutrient content of potato genotypes [8,18,29]. Therefore, the objectives of this research are (1) to assess the magnitude of genetic variability for minerals Fe and Zn; (2) to determine the association between the traits and direct and indirect effects of characters on Fe and Zn; and (3) to identify potential parents for developing varieties with superior Fe and Zn concentrations by using cluster and principal component analyses.

## 2. Result

### 2.1. Analysis of Variance

The results of the analysis of variance revealed the presence of highly significant (*p* ≤ 0.01) differences among the 45 potato genotypes studied in all traits, including Fe and Zn concentrations (Table 1). Average tuber number varied from 2.67 to 16.19, and average tuber weight ranged from 12.55 to 44.70 gm/tuber, whereas tuber yield varied from 0.28 to 2.26 kg/m^2^. Tuber quality traits, dry matter content, specific gravity, and total starch content ranged from 22.15 to 27.93%, 1.086 to 1.113 g cm^−3^, and 15.74 to 20.89%, respectively, whereas Fe and Zn concentrations in tuber dry-weight bases varied from 14.68 to 26.07 mg kg^−1^ and 10.22 to 20.59 mg kg^−1^, respectively (Table 2). Therefore, main tuber yield components, tuber quality, and micronutrients could be considered as selection criteria for the identification of superior promising genotypes for exploitation in future breeding programs. This result agreed with [8,18,30] for Fe and Zn concentrations.

### 2.2. Phenotypic (PCV) and Genotypic (GVC) Coefficients of Variation

Phenotypic variance (𝜎^2^p) and phenotypic coefficient variation (PCV) were higher than genetic variance (𝜎^2^g) and genotypic coefficient of variation (GCV) for all traits, confirming the larger influence of the environment on the expression of these characters (Table 3). The difference between PCV and GCV was relatively high for average tuber weight, average tuber number, total tuber yield, and Fe micronutrients, suggesting that these traits are substantially influenced by the environment. GCV and PCV values ranged from 0.53% to 45.15% and 0.67% to 51.35% for specific gravity and tuber yield, respectively. Deshmukh et al. [31] suggested that PCV and GCV values greater than 20% are considered as high, values between 10% to 20% as medium, and values less than 10% are as low. Accordingly, the highest GCV and PCV values were recorded for average tuber number, average tuber weight, and tuber yield, while medium GCV and PCV were recorded for dry matter content, specific gravity, and starch content. On the other hand, the lowest GCV and PCV were recorded for Fe and Zn micronutrients. A similar study conducted by Amoros et al. [32] showed higher phenotypic variance and phenotypic coefficient of variance than genetic variance and genotypic coefficient of variance for agronomic, yield, and micronutrient traits in diploid potato clones. Similar higher estimates of phenotypic variance than genotypic variance results for yield and processing quality traits were reported by Seid et al. [33]. High GCV and PCV were observed for average tuber number and average tuber weight [34,35,36,37].

### 2.3. Broad-Sense Heritability and Genetic Advance

The genotypic coefficient of variation delivers information about the genetic variability in quantitative traits, but it does not give any estimation about what amount of variation from the genotypic coefficient of variation is heritable. The genetic coefficient of variance together with heritability estimates gives the best picture of the amount of advance to be expected from the selection [38]. Heritability values are useful in predicting the expected advance as percent of mean that ranged from 40.20% for Fe to 77.31% for tuber yield and 0.86% for specific gravity to 81.89% for tuber yield, respectively (Table 3). Broad-sense heritability is categorized as high (>60%), moderate (30–60%), and low (0–30%), and genetic advance as percent of mean is categorized as high (>20%), moderate (10 to 20%), and low (0 to 10) [39]. Accordingly, the highest heritability was observed for tuber yield, average tuber number, Zn micronutrient, dry matter content, starch content, and specific gravity, while moderate heritability was observed for average tuber weight and Fe concentration.

The amount of genetic improvement that would result from selecting individual genotypes is not provided by the heritability value alone. Information about heritability along with genetic advance is more useful. Genetic advance as percent of mean (GAM) was estimated to determine the relative merits of different characters that could be further utilized in the selection of traits in a crop improvement program. A high GAM was obtained from average tuber number, average tuber weight, tuber yield, and zinc content, while a moderate GAM was obtained for Fe content (Table 3). In the present study, high heritability estimates along with high genetic advance as percent of mean were obtained for average tuber number, tuber yield, and Zn concentration. The broad-sense heritability (H^2^) for Fe and Zn concentrations found in the tetraploid population is lower than that reported for Fe (81%) and Zn (81%) in diploid potato clones [32]. The high value of broad-sense heritability (H^2^) estimated for average tuber number per plant, dry matter, starch, and specific gravity contents of potato tubers in the present study is in line with the findings of [22,30,32]. A similar result was reported by Asfaw et al. [8], who recorded high heritability for total tuber yield, marketable tuber yield, and Zn concentration and low heritability for Fe micronutrients.

### 2.4. Phenotypic and Genotypic Correlation of Iron (Fe) and Zinc (Zn) with Other Traits

Table 4 shows the correlation between yield and yield components and micronutrient concentration traits of the potato genotypes evaluated in the present study. Fe concentration had a negative and highly significant correlation with average tuber number, average tuber weight, tuber yield, starch content, dry matter content, and specific gravity at both phenotypic and genotypic levels. Contrarily, Fe concentration had a positive and significant correlation at the genotypic level and a positive and highly significant correlation at the phenotypic level with Zn concentration (Table 4). Zn concentration had a negative and highly significant phenotypic correlation with average tuber number and tuber yield. Furthermore, Zn had a negative and significant correlation with dry matter content, specific gravity, and starch content at the genotypic level. Similarly, Zn had a negative and highly significant phenotypic correlation with all traits except Fe concentration. The correlation between Fe and Zn concentrations was significant and positive (r = 0.68 and *p* < 0.05) at the genotypic level and also highly significant and positive (r = 0.68 and *p* < 0.01) at the phenotypic level. These positive correlations indicate the simultaneous selection for an enhanced level of both micronutrient minerals.

### 2.5. Genotypic Path Coefficient Analysis of Iron (Fe) and Zinc (Zn) Concentration with Other Traits

The results of path analysis at the genotypic level are presented in Table 5. Zn concentration exerted positive direct effects on Fe. The five categories of direct and indirect effects are based on a range of values, viz., negligible (0.00–0.09), low (0.10–0.19), moderate (0.20–0.29), high (0.30–1.00), and very high (>1.00), as Lenka and Mishra [40] reported. According to these groups, genotypic path coefficient analysis revealed that Zn concentration had high and positive direct effects on Fe. Average tuber number exerted moderate and negative direct effects on Fe concentration, whereas average tuber weight had high and negative, tuber yield had negligible and negative, and dry matter content had low and negative direct effects on Fe concentration. Zn had a positive correlation and direct effects on Fe, which suggested that the traits were good contributors and significantly help as selection criteria in potato breeding programs to develop biofortified varieties (Table 5). Genotypic path coefficient analysis showed that Fe content exerted high positive direct effects on Zn. However, average tuber number had moderate negative direct effects and tuber yield and dry matter content had negligible negative direct effects on Zn (Table 6). Zn concentration had weak negative direct effects on total tuber number per plant, average tuber weight, and dry matter content [32].

### 2.6. Principal Component Analysis (PCA)

Principal component analysis with eigenvalues > 1 contributed 76.90% of the total cumulative variance among the 45 potato genotypes. According to eigenvector analysis, the observed variations for the first and second principal components were about 53.60 and 23, respectively (Table 7). In the first principal component analysis, dry matter content (0.41) specific gravity (0.41), starch content (0.41), and average tuber number (0.32) were the most contributing traits, whereas average tuber (0.46) weight (0.46) and tuber yield (0.43) were the highest contributing traits in the second principal component. In another study, the first two PCs accounted for 58.47% of the total cumulative variance observed among the 49 potato genotypes [41].

### 2.7. Cluster Analysis

**Figure 1:**
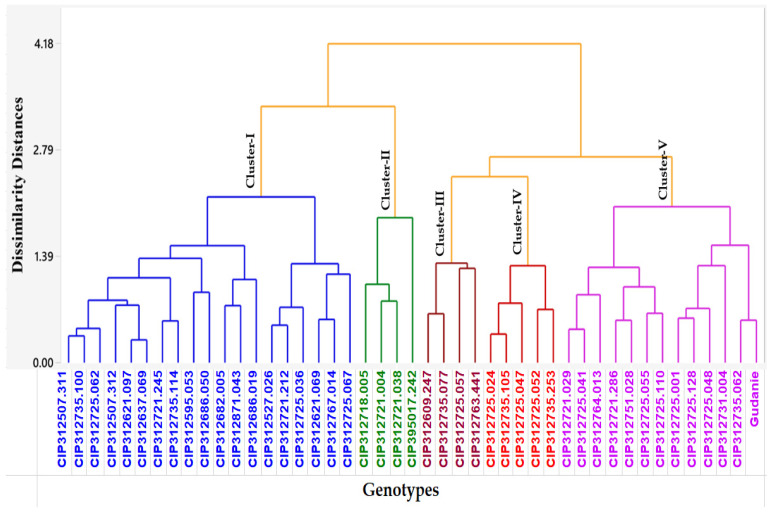
Dendrogram of 45 biofortified potato genotypes classified according to micronutrient, quality, and tuber yield traits.

The Euclidean or genetic distance (degree of dissimilarity) among the 45 potato genotypes studied is presented by the dendrogram cluster analysis tree chart (Figure 1). Cluster I was the largest group, having 19 (42.22%) potato genotypes. This cluster was characterized by high specific gravity. In contrast, Clusters II and III were the smallest, with four (8.89%) potato genotypes. Cluster II represents the most prominent cluster, having better genotype mean values as compared to the mean value of all genotypes for micronutrient concentration traits, viz., Fe (23.80) and Zn (17.07) (Table 8). Five genotypes were categorized under Cluster IV, accounting for 11.11% of total genotypes. The average value of genotypes in the cluster for average tuber number, dry matter content, specific gravity, and starch content are the highest cluster mean values when compared to other cluster genotype means. Cluster V was the second largest group, having 13 (28.89%) potato genotypes, and contained the highest cluster means values for average tuber weight, tuber yield (m^2^), and specific gravity.

## 3. Discussion

The newly introduced and evaluated genotypes in this study provided baseline information that could simplify the decision for releasing improved biofortified potato varieties in Ethiopia. The observed highly significant (*p* ≤ 0.01) variation in micronutrient concentrations and tuber yield traits among the 45 potato genotypes is presented in Table 1. Insights obtained from this study provide a good opportunity for potato breeders to select genotypes with better Fe and Zn concentrations to be used either for developing variety or genotypes that can be used as future parental lines for the targeted traits. Genotype CIP312767.014 has 26.07 mg kg^−1^ Fe and 19.14 mg kg^−1^ Zn, which is 50% more Fe than the control variety ‘Gudanie’ (Table 2).

There were significant differences in agronomic, Fe, and Zn micronutrient traits among widely grown farmer varieties in Ethiopia and introduced potato genotypes from the International Potato Center (CIP) [8]. In a similar study, Burgos et al. [21] reported Fe concentration levels that ranged from 9.4 to 36.7 mg kg^−1^ and Zn concentrations that ranged from 8.3 to 20.2 mg kg^−1^ among 49 different-background potato genotypes on a dry-weight basis. Likewise, Brown et al. [22] reported a wide range in Fe micronutrient content, i.e., 17 to 64 mg kg^−1^, between 33 potato genotypes grown over three locations, portraying the presence of significant differences between genotypes as well as locations. Tuber Zn concentration ranged from 12.5 to 16.1 mg kg^−1^ [30], while Tesfaye et al. [18] reported a much wider concentration range for Fe, from 17.13 to 164.83 mg kg^−1^ and Zn from 7.07 to 20.21 mg kg^−1^, for 21 potato varieties grown over two locations in Ethiopia on a dry-weight basis.

Additional mineral concentration studies carried out on potato by [14,15,16,17] have revealed variability in the Fe concentration of potato tubers ranging from 30 to 185, 11.71 to 131.05, 29.87 to 157.96, and 48.85 to 122.69 mg kg^–1^, and also [29] 92.7 to 96.3 ppm, respectively, on a dry-weight basis. The range in Fe concentration of the 45 genotypes in the current study (from 14.68 to 26.07 mg kg^−1^) and in Zn (from 10.22 to 20.59 mg kg^−1^) agrees with earlier authors’ reports, which reported the presence of substantial genetic variation in the concentrations of Fe and Zn of potato tubers. The differences in the values reported by the different authors from different sets of genotypes studied under different sets of environments (soil fertility gradients, moisture regimes, photoperiods, etc.) is a common observation for many characters as environmental factors influence crop variety performances differently pertaining to the prevailing set of environmental conditions in each set of sampled environments [42].

Soil fertility difference between the trial sites has a substantial effect on the mineral accumulation of plants, which is determined by the phytoavailability of nutrients within the soil and the variation in nutrient uptake and use efficiency [43]. As Bradshaw et al. [19] pointed out, the observed result is a promising opportunity for breeding programs in Ethiopia to increase the bioavailable Fe and Zn concentrations of potato tubers as the heritability of Fe concentration in potato is moderately high. Studies such as [33,44,45,46] reported highly significant differences among genotypes for phenology, tuber yield, and processing quality traits.

Tuber Fe and Zn concentrations indicated a negative and significant correlation and negative direct effect with tuber yield and yield-related traits both at genotypic and phenotypic levels (Table 4, Table 5 and Table 6). This means genotypes with higher concentrations of Fe and Zn did not have higher yields. However, the Fe micronutrient relationship and positive direct effects with Zn concentration could allow the simultaneous improvement for both traits (Table 4, Table 5 and Table 6). The association between Fe and Zn micronutrient concentrations and tuber yield traits was negative, and the correlations between Fe and Zn concentrations were positive and significant [8]. The correlation between Fe and Zn concentrations was weak but positive on a dry-weight basis; however, these correlations were significantly and positively correlated on fresh-weight bases [21]. The correlation between Fe and Zn concentrations was strong and positively significant [18]; however, dry matter content and total tuber yield had a negative association with Fe and Zn concentration. Zn concentration had a positive correlation with Fe and a weak negative direct effect on average tuber number, average tuber weight, and dry matter content [32].

According to cluster analysis, Cluster I consisted of 42.22%, Cluster II and III 8.89%, Cluster IV 11.11%, and Cluster V 28.89% of potato genotypes (Figure 1). A similar trend was observed in cluster analysis among 24 potato genotypes grouped into 8 clusters based on tuber processing quality, yield, and yield-related traits [33]. The 49 potato genotypes were grouped into 2 clusters based on biofortified, yield, and late blight-tolerant traits [41].

## 4. Materials and Methods

### 4.1. Experimental Site, Materials, and Design

The experiment was conducted at the Holetta Agricultural Research Center experiment station located at 09°00′ N, 38°30′ E at an altitude of 2400 m.a.s.l during the main cropping season of 2019. A total of 45 potato genotypes introduced by the International Potato Center (CIP), including the variety ‘Gudanie’, were used for the study (Table 9). These genotypes were selected from the biofortified gene pool for Fe and Zn. The experiment was laid out in a 9 × 5 alpha lattice design with three replications. The plots included 10 plants with a 0.3 m spacing among them and a 0.75 m spacing between rows. A fungicide was used against late blight to protect against the effect of the disease on the tuber yield of the evaluated genotypes. 

### 4.2. Data Collection

All relevant data on tuber yield and yield components, such as average tuber number per hill, average tuber weight (g/tuber), tuber internal and nutritional quality traits such as specific gravity, dry matter, and total raw starch content, as well as iron (Fe) and zinc (Zn) concentration data, were collected following the standard procedures [47,48].

### 4.3. Micronutrient Analysis

#### Sampling and Iron and Zinc Analysis

During harvest, 8 representative tubers of each genotype from each replication were taken for Fe and Zn analysis. Collected tuber samples were thoroughly washed from all observed soils and cleaned with distilled water before peeling and drying for milling. Cleaned and dried samples were milled to powder for mineral analysis according to the procedure described in [48]. Sample tubers were first cleaned from adhered soils and dust before peeling, and rewashed thoroughly with tap water, rinsed with deionized, distilled water, and patted dry with paper towels. Then, tubers were cut longitudinally into four sections and several slices from two opposite end sections of each tuber. A 50 g amount of slices was dried at 80 °C for 48 h until the dried samples had less than 3% moisture content [48]. Once the samples were dried, they were milled, stored in Whirl-Pak plastic bags at room temperature, then sent to Peru for Fe and Zn analysis. Milled tuber samples (3–4 g) were scanned by XRF, as described in [49]. An X-Supreme 8000 (Oxford Instruments) and an energy-dispersive XRF spectrometer equipped with a tungsten X-ray tube, fitted with a 10-place auto-sampler holding 40 mm Al cups, were used. The scans were conducted in Al cups lined with 30 mm polypropylene inner cups sealed at one end with 4 µm thick Poly-4-resistant film using different measurement conditions for Fe and a detection time of 100 s for each element. Fe and Zn concentrations were expressed in mg kg^−1^ dry weight. The elemental concentrations were measured in a set of calibration standards using a reference method by inductively coupled plasma–optical emission spectrophotometry (ICP–OES), and these values were related to the intensity of X-ray emissions for these samples. Fe and Zn concentrations were expressed in mg kg^−1^ dry weight. Since they are found in high levels in soil, dust, or deteriorating laboratory equipment and in low levels in crops, aluminum (Al), titanium (Ti), and chromium (Cr), they are used as indicators of sample contamination. Samples with high Fe levels were analyzed by ICP–OES to give an indication of contamination from the environment, and those with Al > 4 mg kg^−1^, Ti > 0.1 mg kg^−1^, or Cr > 0.2 mg kg^−1^ were eliminated from the data sets to avoid reporting false levels of Fe [49].

### 4.4. Data Analysis

#### 4.4.1. Analysis of Variance

Data were analyzed by restricted maximum likelihood (REML) to fit a mixed model with genotypes, replications, and blocks with replication as random effects. The REML model produced the best linear unbiased predictors (BLUPs), which is a standard method for estimating the random effects of a mixed model. The analysis of variance of yield, quality, and micronutrient concentration traits was performed using the GLM procedure and Tukey for the mean separation trait values of the genotypes using SAS statistical software version 9.3 [50].

Yijk=μ+gi+rj+bkj+Σijk

where Yijk = response of Y trait from the i^th^ genotype, grown in the k^th^ incomplete block of j^th^ replicate, 𝜇 = general mean, gi = random effect of the i^th^ genotype, rj = random effect of the j^th^ replicate, bkj = random effect of k^th^ incomplete block in a j^th^ replicate, and ijk = experimental error. 

#### 4.4.2. Phenotypic and Genotypic Variance

The phenotypic and genotypic variance and coefficients of variance of each trait were calculated using the formula suggested by [51], as follows.

Genotypic variance (σ^2^g)$=\frac{MSg-MSe}{r}$, where σ^2^g = genotypic variance, MSg = mean square of genotypes, MSe = mean square of experimental error, r = number of replications, and environmental variance (𝜎^2^e) = mean square of error.

Phenotypic variance (σ^2^p) = σ^2^g + σ^2^e, where σ^2^p, σ^2^g and σ^2^e = phenotypic, genotypic, and environmental variance, respectively.

$$PCV=\left(\frac{\sqrt{\sigma^{2}p}}{\bar{x}}\right)*100\;\mathrm{and}\;GCV=\left(\frac{\sqrt{\sigma^{2}g}}{\bar{x}}\right)*100$$

where PCV and GCV = phenotypic and genotypic coefficient of variation, respectively, and $\bar{x}$ = grand mean of the character evaluated.

#### 4.4.3. Heritability

Broad-sense heritability (H^2^) is the proportion of phenotypic variance (σ^2^p) and genotypic variance (σ^2^g) using the formula suggested by [52], as follows:

$$H^{2}=(\frac{\sigma 2g}{\sigma 2p})*100$$

#### 4.4.4. Expected Genetic Advance under Selection (GA) and as Percent of Mean (GAM)

Genetic advance in absolute unit (GA) and percent of the mean (GAM), assuming selection of the superior 5% of the genotypes, were estimated in accordance with the methods illustrated by [39], as follows:

GA = K ∗ SDp × H^2^ where, GA = genetic advance, SDp = phenotypic standard deviation on mean basis, H^2^ = broad-sense heritability, and K = standardized selection differential at 5% selection intensity (K = 2.063)

GAM = $\frac{GA}{\bar{X}}$ ∗ 100, where GAM = genetic advance as percent of mean, GA = genetic advance, and $\bar{x}$ = grand mean of the character evaluated.

#### 4.4.5. Phenotypic and Genotypic Correlation Coefficient

Phenotypic (r_p_) and genotypic (r_g_) correlations between two traits were estimated using the formula suggested by [39,51].

$$r_{p}=\frac{{\mathrm{Pcov}}_{\mathrm{xy}}}{\sqrt{\left(V_{p}x{\cdot V}_{p}y\right)}}$$

$$r_{g}=\frac{{\mathrm{Gcov}}_{\mathrm{xy}}}{\sqrt{\left(V_{g}x{\cdot V}_{g}y\right)}}$$

where r_p_ and r_g_ = phenotypic and genotypic correlation coefficient, Pcov_xy_ and Gcov_xy_ = phenotypic and genotypic covariance between variables x and y, V_p_x and V_g_x = phenotypic and genotypic variance of variable x, and V_p_y and V_g_x = phenotypic and genotypic variance of variable y, respectively. The calculated phenotypic correlation value was tested for its significance using *t*-test: t = r_ph_/SE (r_p_), where r_p_ = phenotypic correlation, and SE (r_p_) = standard error of phenotypic correlation obtained using the following formula of [25].

$$SE(rp)=\sqrt{\frac{1-r2ph}{n-2}}$$

where n is the number of genotypes tested, and r^2^_p_ is the phenotypic correlation coefficient.

The coefficient of correlations at genotypic levels was tested for their significance by the formula described by [53], as indicated below: t = r_gxy_/Ser_gxy_

#### 4.4.6. Path Coefficient Analysis

Path coefficient analysis was calculated to partition the correlation coefficient to direct and indirect effects of the characters on Fe and Zn concentrations as suggested by [54] using the formula rij = Pij + ∑rikpkj, where rij = correlation between the independent trait (i) and dependent trait (j), as measured by the genotypic and phenotypic correlation coefficients; Pij = direct effects of the independent trait (i) on the dependent trait (j), as measured by the genotypic path coefficients; and Σrikpkj = summation of components of indirect effects of a given independent trait (i) on a given dependent trait (j) by all other independent traits (k).

#### 4.4.7. Principal Component Analysis (PCA)

Principal component analysis was computed from average tuber number, average tuber weight, tuber yield, dry matter content, specific gravity, total starch content, and Fe and Zn concentration levels to find out if the traits accounted more for the total variation. The data were standardized to the mean zero and variance of one before being computed by principal component analysis. PCA based on the correlation matrix was calculated using SAS software. According to Gutten’s lower-bound principle, eigenvalues < 1 should be ignored [55].

#### 4.4.8. Genetic Distance and Clustering

The genetic distance of 45 potato genotypes was estimated using the Euclidean distance (ED) calculated from tuber yield, quality, and micronutrients concentration traits after subtracting the mean value and dividing it by the standard deviation established by [56], as follows:

$$\mathrm{EDjk}=\overset{2}{\sqrt{\sum_{i=1}^{n}{\left(Xij-Xik\right)}^{2}}}$$

where EDjk = distance between genotypes j and k; xij and xik = phenotype traits values of the i^th^ characters for genotypes j and k, respectively; and n = number of phenotype traits used to calculate the distance. The distance matrix from phenotype traits was used to construct a dendrogram based on the unweighted pair-group methods with arithmetic means (UPGMA). The results of cluster analysis are presented in the form of a dendrogram.

## 5. Conclusions

This research revealed a significant genetic diversity and positive correlation for Fe and Zn concentrations in potato genotypes, which may allow for the directed selection of parents in future breeding programs in Ethiopia. Fe and Zn concentrations showed a significant negative correlation and direct effects. This might lead to difficulties in simultaneous selection for the increased tuber yield and concentrations of both minerals. According to cluster mean analysis, Cluster II contained the best genotypes for higher Fe and Zn concentrations. It could be used for further selection and also included as parents in crossing programs targeting the development of biofortified potato varieties. In conclusion, it is advisable to proceed with further study on more locations, including more genotypes, to identify several potato genotypes with more locations suitable for breeding or as parents for generating new genotypes with high Fe and Zn contents considering good tuber yield.

## Figures and Tables

**Table 1:** Expected mean squares of yield component, quality, and mineral traits of potato genotypes grown at Holetta, 2019.

Source of Variation	DF	Traits							
		AvTN	AvTW (g/tuber)	TY (kg/m^2^)	DMC (%)	SG (g cm^−3^)	TSC (g/100 g)	Fe (mg kg^−1^)	Zn (mg kg^−1^)
Replication	2	11.33	84.35	0.36	1.41	0.00003	1.13	98.75	59
Block	24	4.37	67.82	0.12	1.73	0.00004	1.37	10.18	1.95
Genotype	44	33.23 **	165.24 **	0.73 **	5.84 **	0.00012 **	4.63 **	20.69 **	13.48 **
Error	64	4.39	43.73	0.07	0.96	0.00002	0.76	6.86	2.06
Mean		8.36	26.94	1.04	26.25	1.105	19.39	20.59	14.6
CV (%)		25.08	24.55	24.46	3.74	0.41	4.51	12.72	9.84

DF—degree of freedom; AvTN—average tuber number; AvTW—average tuber weight; TY—tuber yield; DMC—dry matter content; SG—specific gravity; TSC—total starch content; Fe—iron; Zn—zinc; ** significant at *p* < 0.01.

**Table 2:** Mean values of agronomic, quality, and nutrient content traits of the 45 potato genotypes grown at Holetta, 2019.

Genotypes	AvTN	AvTW (g/tuber)	TY (kg/m^2^)	DMC (%)	SG (g cm^−3^)	STC (g/100 g)	Fe (mg kg ^−1^)	Zn (mg kg ^−1^)
CIP312507.311	6.53 ^g–l^	28.26 ^a–e^	0.81 ^f–o^	27.09 ^ab^	1.109 ^ab^	20.15 ^ab^	22.03 ^a–e^	14.73 ^b–j^
CIP312507.312	6.66 ^g–l^	21.75 ^b–e^	0.76 ^g–o^	27.31 ^a^	1.110 ^a^	20.34 ^a^	19.92 ^a–e^	14.21 ^d–j^
CIP312527.026	3.63 ^j–l^	18.12 ^c–e^	0.29 ^no^	25.22 ^a–e^	1.101 ^a–e^	18.48 ^a–e^	24.44 ^a–d^	15.38 ^b–i^
CIP312595.053	2.67 ^l^	28.38 ^a–e^	0.36 ^m–o^	25.97 ^a–d^	1.104 ^a–d^	19.14 ^a–d^	19.74 ^a–e^	13.05 ^e–j^
CIP312609.247	8.34 ^c–l^	34.17 ^a–e^	1.60 ^a–g^	25.11 ^a–e^	1.100 ^a–e^	18.38 ^a–e^	17.71 ^a–e^	12.93 ^e–j^
CIP312621.069	5.33 ^h–l^	19.60 ^c–e^	0.53 ^k–o^	25.40 ^a–e^	1.101 ^a–e^	18.64 ^a–e^	25.40 ^ab^	20.59 ^a^
CIP312621.097	7.21 ^e–l^	30.43 ^a–e^	0.96 ^e–o^	27.54 ^a^	1.111 ^a^	20.54 ^a^	20.77 ^a–e^	14.49 ^c–j^
CIP312637.069	6.98 ^f–l^	34.04 ^a–e^	1.03 ^e–o^	27.35 ^a^	1.110 ^a^	20.37 ^a^	20.41 ^a–e^	13.41 ^e–j^
CIP312682.005	6.93 ^f–l^	18.57 ^c–e^	0.51 ^l–o^	26.81 ^a–c^	1.108 ^a–c^	19.89 ^a–c^	22.66 ^a–e^	16.36 ^a–g^
CIP312686.019	5.21 ^h–l^	15.04 ^de^	0.42 ^l–o^	26.97 ^a–c^	1.109 ^a–c^	20.04 ^a–c^	22.30 ^a–e^	11.32 ^h–j^
CIP312686.050	4.50 ^i–l^	23.87 ^a–e^	0.48 ^l–o^	26.12 ^a–d^	1.105 ^a–d^	19.28 ^a–d^	18.40 ^a–e^	16.83 ^a–f^
CIP312718.005	4.37 ^i–l^	31.35 ^a–e^	0.61 ^i–o^	22.15 ^e^	1.086 ^e^	15.74 ^e^	24.03 ^a–d^	18.78 ^a–d^
CIP312721.004	4.69 ^i–l^	30.83 ^a–e^	0.67 ^h–o^	23.18 ^de^	1.091 ^de^	16.65 ^de^	24.99 ^a–c^	15.24 ^b–i^
CIP312721.029	8.74 ^b–l^	33.03 ^a–e^	1.67 ^a–f^	26.99 ^a–c^	1.109 ^a–c^	20.05 ^a–c^	16.74 ^b–e^	12.78 ^f–j^
CIP312721.038	5.02 ^i–l^	22.50 ^a–e^	0.43 ^l–o^	22.15 ^e^	1.087 ^e^	15.74 ^e^	24.78 ^a–d^	14.79 ^b–j^
CIP312721.212	3.86 ^j–l^	18.30 ^c–e^	0.30 ^no^	25.48 ^a–d^	1.102 ^a–d^	18.71 ^a–d^	22.61 ^a–e^	16.94 ^a–f^
CIP312721.245	5.99 ^g–l^	26.28 ^a–e^	0.76 ^g–o^	27.12 ^ab^	1.109 ^ab^	20.17 ^ab^	23.95 ^a–d^	17.74 ^a–e^
CIP312721.286	7.42 ^d–l^	35.18 ^a–d^	1.15 ^d–n^	27.61 ^a^	1.112 ^a^	20.61 ^a^	19.47 ^a–e^	12.63 ^f–j^
CIP312725.001	14.16 ^a–e^	32.64 ^a–e^	2.13 ^ab^	27.66 ^a^	1.112 ^a^	20.65 ^a^	17.67 ^a–e^	12.49 ^f–j^
CIP312725.024	13.85 ^a–f^	14.33 ^de^	1.01 ^e–o^	26.86 ^a–c^	1.108 ^a–c^	19.94 ^a–c^	19.90 ^a–e^	13.35 ^e–j^
CIP312725.036	6.07 ^g–l^	20.42 ^b–e^	0.54 ^j–o^	24.84 ^a–e^	1.099 ^a–e^	18.14 ^a–e^	21.70 ^a–e^	14.85 ^b–j^
CIP312725.041	11.03 ^a–i^	31.32 ^a–e^	1.52 ^a–h^	26.53 ^a–c^	1.107 ^a–c^	19.64 ^a–c^	17.62 ^a–e^	13.13 ^e–j^
CIP312725.047	15.68 ^ab^	18.25 ^c–e^	1.28 ^b–l^	26.92 ^a–c^	1.108 ^a–c^	19.99 ^a–c^	21.52 ^a–e^	13.71 ^e–j^
CIP312725.048	12.13 ^a–h^	33.67 ^a–e^	1.82 ^a–e^	27.23 ^ab^	1.110 ^a^	20.27 ^ab^	19.39 ^a–e^	14.64 ^b–j^
CIP312725.052	14.36 ^a–d^	18.49 ^c–e^	1.06 ^d–o^	27.93 ^a^	1.113 ^a^	20.89 ^a^	18.23 ^a–e^	12.38 ^f–j^
CIP312725.055	8.54 ^c–l^	28.41 ^a–e^	1.44 ^a–i^	27.24 ^a^	1.110 ^a^	20.27 ^a^	19.83 ^a–e^	15.73 ^a–i^
CIP312725.057	11.14 ^a–i^	25.08 ^a–e^	1.26 ^c–l^	23.92 ^b–e^	1.095 ^b–e^	17.32 ^b–e^	22.45 ^a–e^	12.44 ^f–j^
CIP312725.062	5.88 ^g–l^	30.06 ^a–e^	0.78 ^f–o^	26.34 ^a–d^	1.106 ^a–d^	19.47 ^a–d^	21.81 ^a–e^	14.25 ^d–j^
CIP312725.067	10.55 ^a–j^	15.94 ^de^	0.73 ^h–o^	24.94 ^a–e^	1.099 ^a–e^	18.22 ^a–e^	24.65 ^a–d^	17.10 ^a–f^
CIP312725.110	9.41 ^a–l^	32.47 ^a–e^	1.69 ^a–e^	27.78 ^a^	1.112 ^a^	20.75 ^a^	21.60 ^a–e^	14.99 ^b–j^
CIP312725.128	12.17 ^a–h^	32.44 ^a–e^	1.76 ^a–e^	27.53 ^a^	1.111 ^a^	20.53 ^a^	19.47 ^a–e^	11.73 ^g–j^
CIP312731.004	16.19 ^a^	28.47 ^a–e^	2.03 ^a–c^	26.06 ^a–d^	1.104 ^a–d^	19.22 ^a–d^	16.07 ^de^	11.42 ^h–j^
CIP312735.062	9.86 ^a–k^	42.79 ^ab^	2.26 ^a^	26.52 ^a–c^	1.107 ^a–c^	19.63 ^a–c^	16.53 ^c–e^	11.14 ^ij^
CIP312735.077	11.03 ^a–i^	30.50 ^a–e^	1.49 ^a–h^	25.83 ^a–d^	1.103 ^a–d^	19.02 ^a–d^	18.94 ^a–e^	14.75 ^b–j^
CIP312735.100	6.42 ^g–l^	26.12 ^a–e^	0.74 ^g–o^	26.42 ^a–d^	1.106 ^a–c^	19.54 ^a–d^	22.36 ^a–e^	16.13 ^a–h^
CIP312735.105	12.58 ^a–g^	12.55 ^e^	0.71 ^h–o^	27.22 ^ab^	1.109 ^ab^	20.25 ^ab^	19.77 ^a–e^	13.93 ^d–j^
CIP312735.114	7.07 ^f–l^	29.57 ^a–e^	1.06 ^d–o^	27.52 ^a^	1.111 ^a^	20.52 ^a^	23.23 ^a–e^	15.78 ^a–i^
CIP312735.253	14.56 ^a–c^	20.86 ^b–e^	1.38 ^b–k^	27.65 ^a^	1.112 ^a^	20.64 ^a^	16.43 ^c–e^	11.25 ^ij^
CIP312751.028	7.65 ^c–l^	39.84 ^a–c^	1.40 ^a–j^	27.90 ^a^	1.113 ^a^	20.86 ^a^	18.26 ^a–e^	14.40 ^c–j^
CIP312763.441	8.72 ^b–l^	18.93 ^c–e^	0.73 ^h–o^	24.84 ^a–e^	1.099 ^a–e^	18.14 ^a–e^	17.69 ^a–e^	13.91 ^e–j^
CIP312764.013	10.33 ^a–k^	30.69 ^a–e^	1.20 ^c–m^	26.90 ^a–c^	1.108 ^a–c^	19.98 ^a–c^	17.49 ^a–e^	16.63 ^a–f^
CIP312767.014	5.58 ^g–l^	25.32 ^a–e^	0.66 ^h–o^	25.39 ^a–e^	1.101 ^a–e^	18.63 ^a–e^	26.07 ^a^	19.14 ^a–c^
CIP312871.043	3.31 ^kl^	18.80 ^c–e^	0.28 ^o^	27.24 ^ab^	1.110 ^ab^	20.27 ^ab^	20.79 ^a–e^	15.88 ^a–i^
CIP395017.242	4.23 ^i–l^	39.94 ^a–c^	0.75 ^g–o^	23.71 ^c–e^	1.094 ^c–e^	17.13 ^c–e^	21.80 ^a–e^	19.48 ^ab^
Gudanie	9.40 ^a–l^	44.7 ^a^	1.91 ^a–d^	26.59 ^a–c^	1.107 ^a–c^	19.69 ^a–c^	14.68 ^e^	10.22 ^j^
Range	2.67–16.19	12.55–44.70	0.28–2.26	22.15–27.93	1.086–1.113	15.74–20.89	14.68–26.07	10.22–20.59
Mean	8.35	26.94	1.04	26.25	1.105	19.39	20.59	14.6

AvTN—average tuber number; AvTW—average tuber weight; TY—tuber yield; DMC—dry matter content; SG—specific gravity; TSC—total starch content; Fe—iron; Zn—zinc; mean values with similar letter(s) in each column had non-significant differences between genotypes using Tukey’s test at *p* < 0.05.

**Table 3:** Estimates of variance, coefficients of variation, heritability, genetic advance, and genetic advance as percentage of means values for various traits of potato genotypes grown at Holetta, 2019.

Traits	σ^2^g	σ^2^p	σ^2^e	GCV	PCV	H^2^%	GA (5%)	GAM%	PCV-GCV
AvTN	9.61	14.01	4.39	37.11	44.79	68.64	5.30	63.43	7.68
AvTW (g/tuber)	40.50	84.23	43.73	23.62	34.07	48.09	9.10	33.79	10.44
TY (kg/m^2^)	0.22	0.29	0.07	45.15	51.35	77.31	0.85	81.89	6.20
DMC (%)	1.62	2.59	0.96	4.86	6.13	62.81	2.08	7.94	1.27
SG (g cm^−3^)	0.00003	0.00005	0.00002	0.53	0.67	62.61	0.01	0.86	0.14
TSC (g/100 g)	1.29	2.05	0.76	5.86	7.39	62.80	1.86	9.57	1.53
Fe (mg kg^−1^)	4.61	11.47	6.86	10.43	16.45	40.20	2.81	13.64	6.02
Zn (mg kg^−1^)	3.81	5.87	2.06	13.36	16.59	64.86	3.24	22.20	3.23

𝜎^2^p—phenotypic variance; 𝜎^2^g—genotypic variance; 𝜎^2^e—environmental variance; PCV—phenotypic coefficients of variations; GCV—genotypic coefficients of variations; H^2^—broad-sense heritability; GA—genetic advance; GAM—genetic advance as percentage of means; AvTN—average tuber number; AvTW—average tuber weight; TY—Tuber Yield; DMC—dry matter content; SG—specific gravity; TSC—total starch content; Fe—iron; Zn—zinc.

**Table 4:** Estimates of phenotypic (below diagonal) and genotypic (above diagonal) correlation coefficients among various traits of potato genotypes grown at Holetta, 2019.

Variable	AvTN	AvTW	TY	DMC	SG	TSC	Fe	Zn
Average tuber number		−0.02 ^ns^	0.71 **	0.39 **	0.38 **	0.39 **	−0.54 **	−0.53 **
Average tuber weight (g/tuber)	−0.03 ^ns^		0.57 **	0.06 ^ns^	0.06 ^ns^	0.06 ^ns^	−0.39 **	−0.18 ^ns^
Tuber Yield (kg/m^2^)	0.67 **	0.62 **		0.38 *	0.38 *	0.38 *	−0.68 **	−0.56 **
Dry matter content (%)	0.31 **	0.04 ^ns^	0.30 **		1.00 **	1.00 **	−0.47 **	−0.38 *
Specific gravity (g cm^−3^)	0.31 **	0.04 ^ns^	0.30 **	1.00 **		1.00 **	−0.47 **	−0.38 *
Total starch content (g/100 g)	0.31 **	0.037 **	0.30 **	1.00 **	1.00 **		−0.47 **	−0.38 *
Fe (mg kg^−1^)	−0.27 **	−0.12 *	−0.39 **	−0.36 **	−0.37 **	−0.36 **		0.68 *
Zn (mg kg^−1^)	−0.39 **	−0.09 ^ns^	−0.39 **	−0.32 **	−0.32 **	−0.32 **	0.68 **	

**—correlation is significant at *p* < 0.01; *—correlation is significant at *p* < 0.05; ^ns^—non-significant.

**Table 5:** Estimates of direct (bold) and indirect effect (off-diagonal) of different traits on iron (Fe) at genotypic level in 45 potato genotypes tested at Holetta, 2019.

Variable	AvTN	AvTW	TY	DMV	Zn	r_g_
Average tuber number	**−0.24**	0.01	−0.01	−0.07	−0.23	−0.54 **
Average tuber weight (g/tuber)	0.01	**−0.30**	0.00	−0.01	−0.08	−0.39 **
Tuber Yield (kg/m^2^)	−0.17	−0.19	**−0.01**	−0.07	−0.24	−0.68 **
Dry matter content (%)	−0.09	−0.02	0.00	**−0.19**	−0.16	−0.47 **
Zinc (mg kg^−1^)	0.13	0.05	0.00	0.07	**0.42**	0.68 *

**—correlation is significant at *p* < 0.01; *—correlation is significant at *p* < 0.05; r_g_—genotypic correlation coefficient.

**Table 6:** Estimates of direct (bold) and indirect effect (off-diagonal) of different traits on Zinc (Zn) at genotypic level in 45 potato genotypes tested at Holetta, 2019.

Variable	AvTN	TY	DMC	Fe	r_g_
Average tuber number	**−0.20**	−0.04	−0.01	−0.28	−0.53 **
Tuber Yield (kg/m^2^)	−0.14	**−0.06**	−0.01	−0.35	−0.56 **
Dry matter content (%)	−0.08	−0.02	**−0.04**	−0.24	−0.38 *
Iron (mg kg^−1^)	0.11	0.04	0.02	**0.52**	0.68 *

**—correlation is significant at *p* < 0.01; *—correlation is significant at *p* < 0.05; r_g_—genotypic correlation coefficient.

**Table 7:** Eigenvalue, percentage, and cumulative variances of the first two principal components for eight quantitative traits in potato genotypes.

Variable	PC1	PC2
Average tuber number	0.32	0.18
Average tuber weight (g/tuber)	0.15	0.46
Tuber Yield (kg/m^2^)	0.36	0.43
Dry matter content (%)	0.41	−0.38
Specific gravity (g cm^−3^)	0.41	−0.38
Total starch content (g/100 g)	0.41	−0.38
Fe (mg kg^−1^)	−0.37	−0.29
Zn (mg kg^−1^)	−0.33	−0.24
Eigenvalue	4.29	1.87
Variances (%)	53.60	23.30
Cumulative variances (%)	53.60	76.90

**Table 8:** Cluster mean analysis.

Variable	Cluster 1	Cluster 2	Cluster 3	Cluster 4	Cluster 5
Average tuber number	5.81	4.58	9.81	14.21	10.54
Average tuber weight (g)	23.52	30.72	28.17	16.93	34.25
Tuber yield (kg/m^2^)	0.63	0.60	1.30	1.06	1.69
Dry matter content (%)	26.40	22.77	24.94	27.32	27.08
Specific gravity (g cm^−3^)	1.11	1.09	1.10	1.11	1.11
Starch content (g/100 g)	19.53	16.29	18.23	20.35	20.13
Fe (mg kg^−1^)	22.30	23.80	19.05	19.39	18.01
Zn (mg kg^−1^)	15.69	17.07	13.51	12.93	13.23

**Table 9:** List of potato genotypes from the CIP biofortified breeding gene pool tested at Holetta Agricultural Research Center, Ethiopia 2019.

No.	Genotypes	No.	Genotypes	No.	Genotypes
1	CIP312507.311	16	CIP312721.212	31	CIP312725.128
2	CIP312507.312	17	CIP312721.245	32	CIP312731.004
3	CIP312527.026	18	CIP312721.286	33	CIP312735.062
4	CIP312595.053	19	CIP312725.001	34	CIP312735.077
5	CIP312609.247	20	CIP312725.024	35	CIP312735.100
6	CIP312621.069	21	CIP312725.036	36	CIP312735.105
7	CIP312621.097	22	CIP312725.041	37	CIP312735.114
8	CIP312637.069	23	CIP312725.047	38	CIP312735.253
9	CIP312682.005	24	CIP312725.048	39	CIP312751.028
10	CIP312686.019	25	CIP312725.052	40	CIP312763.441
11	CIP312686.050	26	CIP312725.055	41	CIP312764.013
12	CIP312718.005	27	CIP312725.057	42	CIP312767.014
13	CIP312721.004	28	CIP312725.062	43	CIP312871.043
14	CIP312721.029	29	CIP312725.067	44	CIP395017.242
15	CIP312721.038	30	CIP312725.110	45	Gudanie

CIP—International Potato Center.

## Data Availability

All data are available in the main text.
